# Innovative Gas-Liquid Membrane Contactor Systems for Carbon Capture and Mineralization in Energy Intensive Industries

**DOI:** 10.3390/membranes11040271

**Published:** 2021-04-08

**Authors:** Akrivi Asimakopoulou, Dimitrios Koutsonikolas, Georgia Kastrinaki, George Skevis

**Affiliations:** 1 Centre for Research and Technology Hellas (CERTH), Chemical Process & Energy Resources Institute (CPERI), 57001 Thessaloniki, Greece; dkoutson@certh.gr (D.K.); gskevis@certh.gr (G.S.); 2 Laboratory of Inorganic Materials (LIM), Centre for Research and Technology Hellas (CERTH), Chemical Process & Energy Resources Institute (CPERI), 57001 Thessaloniki, Greece; georgiak@certh.gr

**Keywords:** gas-liquid membrane contactor, hollow fibers, carbon dioxide capture, calcium carbonate, mineralization, mass transfer

## Abstract

CO_2_ mineralization is an alternative to conventional geological storage and results in permanent carbon storage as a solid, with no need for long-term monitoring and no requirements for significant energy input. Novel technologies for carbon dioxide capture and mineralization involve the use of gas-liquid membrane contactors for post-combustion capture. The scope of the present study is to investigate the application of hollow fiber membrane contactor technology for combined CO_2_ capture from energy-intensive industry flue gases and CO_2_ mineralization, in a single-step multiphase process. The process is also a key enabler of the circular economy for the cement industry, a major contributor in global industrial CO_2_ emissions, as CaCO_3_ particles, obtained through the mineralization process, can be directed back into the cement production as fillers for partially substituting cement in high-performance concrete. High CO_2_ capture efficiency is achieved, as well as CaCO_3_ particles of controlled size and crystallinity are synthesized, in every set of operating parameters employed. The intensified gas-liquid membrane process is assessed by calculating an overall process mass transfer coefficient accounting for all relevant mass transfer resistances and the enhanced mass transfer due to reactive conditions on the shell side. The obtained nanocomposite particles have been extensively characterized by DLS, XRD, TGA, SEM, TEM, and FTIR studies, revealing structured aggregates (1–2 μm average aggregate size) consisting of cubic calcite when the contactor mode is employed.

## 1. Introduction

CO_2_ capture and utilization by mineralization (CCUM) is a distinct pathway for carbon capture, utilization, and storage (CCUS) that involves simultaneous CO_2_ capture and chemical conversion into a stable product [1] and is currently considered as a promising technology with significant advantages over CO_2_ capture with conventional technologies (i.e., absorption, adsorption) [2]. During mineralization, an exothermic reaction occurs between captured CO_2_ and cations, such as Ca^2+^ and Mg^2+^, derived from minerals or industrial waste, to produce metal carbonates [3,4]. Mineralization reduces natural resource use and converts waste to added-value products, thus promoting a circular economy [3] through an environmentally benign option [5]. Even though mineralization can reduce waste treatment [6] and potentially store up to 10^4^–10^6^ gigatons of CO_2_ annually [7], there are several challenges associated with conventional technologies that limit its potential large-scale industrial deployment [8]. Although the conversion process is thermodynamically favorable, kinetic limitations, resulting from the low solubility of CO_2_ in water, and the mass transfer limitations of cation dissolution and carbonate precipitation [7,9] have to be overcome. Mechanical, thermal, and/or chemical treatment along with high pressures are needed for reactions to proceed at acceptable rates. [8].

Among various types of mineral carbonation (e.g., [1]), the ex situ direct single-step mineralization pathway is currently gaining increasing attention. In such a process, CO_2_ is captured in a solvent (e.g., ammonium hydroxide (NH_4_OH) [10,11], alkanolamines (monoethanolamine MEA, diethanolamine DEA, methyldiethanolamine MDEA) [12], etc.), and a carbonate mineral is simultaneously produced through reaction with Ca^2+^ ions, present in the same alkaline sorbent, resulting thus in an in-situ, one step, chemical recycling of the solvent. This approach is further advantageous since a single temperature can be used, and both reactions can be accelerated with improved efficiency and ultimately costs [7]. Another promising approach is the direct mineralization under ambient temperatures and pressures in fluidized bed reactors [13]. Packed columns have also received increased attention since the large gas-liquid contact areas that can be attained in them ultimately lead to increased mass transfer and reaction rates [14]. However, they are subject to operating problems like flooding, channeling, foaming, and entrainment and they can also be energy-intensive [14]. 

Furthermore, carbon utilization through the carbonation route is often exploited for the production of calcium carbonate in various continuous or semi-batch-stirred reactors [7,11,15,16,17,18,19], with CaCO_3_ particle size and morphology controlled by operational parameters such as reaction time, temperature, stirring rate, calcium/carbonate ions concentration. The sources of Ca^2+^ can vary broadly, with CaCl_2_ [11,15] and slurry solutions containing Ca(OH)_2_ [17] and CaO [16,18] commonly reported. The source of Ca^2+^ ions in the current process is calcium chloride (CaCl_2_), a by-product of the Solvay process used for the production of sodium carbonate. Carbon mineralization by CaCl_2_ as a precursor under alkaline conditions (pH > 12) is widely studied in the literature and is expected to yield calcite crystalline forms [12]. Depending on the initial solution temperature and supersaturation level other specific crystalline forms can be obtained. Calcium carbonate nanoparticles can also be derived from saturated sodium carbonate and calcium nitrate aqueous solutions, where aragonite precipitation predominates [20].

**Figure 1:**
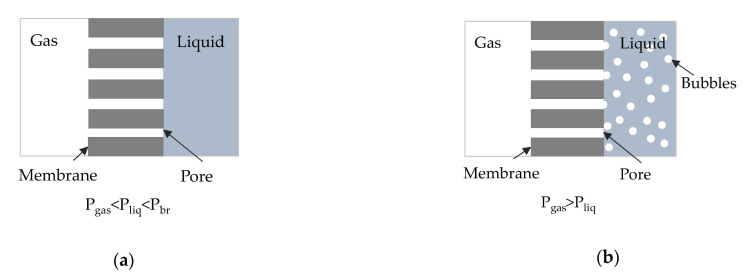
Cross section of a hydrophobic membrane (**a**) in contactor mode, (**b**) in bubbling mode.

Novel technologies for CCUM involve the use of gas-liquid membrane contactors for post-combustion capture. Hollow fiber membrane contactors are well established in the field of gas separation/bubbling/extraction applications since very large and well-defined surface areas can be obtained in hyper compact membrane modules [21,22]. Using a hydrophobic microporous membrane two different modes of operation can be identified depending on operating pressure (Figure 1): (a) the membrane contactor mode (Figure 1a), in which an immobilized gas-liquid interface is formed at the pores’ mouth in the liquid side, by keeping the liquid pressure higher than the gas pressure and lower than the breakthrough pressure, that is, the pressure where liquid enters into the pores of a hydrophobic membrane (P_br_ > P_liq_ > P_gas_) and (b) the membrane bubbling reactor mode, (Figure 1b), in which gas enters in the liquid phase in the form of nano-bubbles, by keeping the gas pressure higher than the liquid pressure (P_gas_ > P_liq_). 

The membrane contactor process has several advantages as the gas-liquid interface lies at the pores’ edge with no dispersion of the gaseous phase into the liquid solvent, and by properly selecting the membrane material and the process parameters, high mass transfer rates can be achieved. The membrane bubbling reactor can also be advantageous since, depending on membrane pore size, distinct nanobubbles can be formed with a high surface area to volume ratio and increased stability thus avoiding collapse or coalesce in the liquid medium compared to the larger size bubbles [23].

Membranes in general have been used in a variety of configurations to produce nanostructured materials [24,25]. Specifically, hollow fiber membrane contactors can be used for direct CO_2_ reactive capture from the flue gases and simultaneous conversion to useful carbonates [26,27,28]. The benefits of employing hollow fiber membrane contactors in this type of application come from their distinct characteristics (i.e., large specific contact area, precisely controlled pore size, etc.) [29,30]. Although only a few experimental studies have been published so far, these revealed that membrane-based precipitation of carbonates offers an ideal route for mineralization with controllable morphological and structural properties of the generated particles.

In the current study, an innovative process based on a gas-liquid membrane contactor system is developed and evaluated for carbon capture and mineralization. Specifically, a polypropylene hollow fiber membrane module was employed for CaCO_3_ production under contactor and/or bubbling mode and the morphological properties of the generated particles were studied. The main process parameters were identified and their effect on the process performance and the characteristics of the generated CaCO_3_ particles was assessed. 

## 2. Materials and Methods 

### 2.1. Materials

In the present study, calcium chloride dihydrate, CaCl_2_·2H_2_O (Scharlab, Sentmenat, Spain, CA0193025P, powder, extra pure, Pharmpur^®^, Königsbrunn, Germany, Ph Eur, BP, USP, 99–103%) and ammonium hydroxide solution NH_4_OH·H_2_O (puriss. p.a., reag. ISO, reag. Ph. Eur., ≥25% NH_3_ basis) were used for the preparation of liquid feed phase. Pure CO_2_ (99.9%) or a binary mixture (CO_2_/N_2_) of 20% CO_2_ (99.9%) and 80% N_2_ (99.999%) were fed as gas phase.

### 2.2. Experimental Setup and Procedure

**Figure 2:**
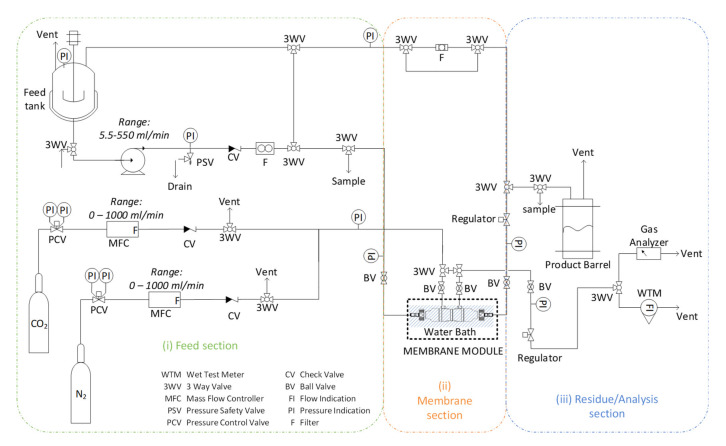
Schematic depiction of the lab-scale membrane reactor/precipitator experimental unit setup.

Α lab-scale membrane reactor/precipitator experimental unit was developed (Figure 2). The experimental unit consists of three different sections: (i) the feed section, (ii) the gas-liquid membrane contactor section, (iii) the residue/analysis section. The unit setup can be operated either with liquid recycle representing a semi-batch operation mode or on a once-through mode representing a continuous operation mode.

(i) Feed section: In a 6 l Stainless Steel (SS316) container vessel equipped with pressure gauge and safety valve, mixing of liquid precursors and preparation of the feed solution takes place: 4.48 g calcium chloride dihydrate is dissolved in 2L of double distilled water and is mixed with 257.6 g aqueous solution of ammonium hydroxide (25% *w*/*w*). Liquid feed phase is being circulated with a pump (Ismatec ISM446B-230V (B-MOUNT) BVP-Z Analog Gear Pump Drive), with a relief valve preset at three bars and a float ball flowmeter (Q_l_ = 0–0.5 l/min). Through a three-way valve, the liquid phase either recirculates into the mixing vessel, or is directed to the membrane section. Gas phase (binary gas mixture (CO_2_/N_2_) or pure CO_2_) is fed at a flowrate (Q_g_ = 0.1–0.5 l/min) controlled by a set of Mass Flow Controllers (MFCs) (Bronkhorst, AK Ruurlo, The Netherlands, F-201CV-20K-AAD-22-V, 1l/min, 5 bar(g)/3 bar(g), CO_2_ and Bronkhorst F-201CV-20K-AAD-22-V, 1l/min, 5 bar(g)/3 bar(g), N_2_). 

(ii) Membrane section: 3M™ Charlotte, USA, Liqui-Cel™ is a leading manufacturer of membrane contactors, which offers a wide range of membrane modules (from lab to commercial scale), designed for mass transfer applications in gas-liquid systems (e.g., adding gases to or removing dissolved gases and bubbles from liquids). These modules have a very large specific surface area, reproducible properties and have been studied extensively in the literature for CO_2_ capture applications. Therefore, a commercial lab-scale membrane module of 3M™ Liqui-Cel™ was selected for the current tests (details in Table 1). The gas and liquid feed phase enter into the fiber lumen and shell side, respectively, in a co-current or counter-current mode of flow direction. Two liquid phase sampling points are enabled by reasonably placed three-way valves at the inlet and outlet of the membrane module. On every side of the membrane module (i.e., entry and exit), pressure is monitored with pressure gauges. Pressure-regulating valves are used at the outlet of both gas and liquid phases to set a constant pressure at the exit of the membrane lumen side, and in addition, with an ON/OFF valve, strangling of the flow can be optionally achieved and thus gas is forced entirely through the pores of the membrane walls, in a bubbling mode of operation. (iii) Residue/Analysis section: The carbonation reaction between CO_2_, CaCl_2_, and NH_4_OH is monitored by measuring the pH of the solution. When no more significant pH changes are observed, both liquid and gas flowrates are zeroed and the test is terminated. Calcium carbonate particles produced are filtered (Sigma Aldrich, Steinheim, Germany, Whatman 589/3 Blue ribbon, ashless filter paper circles) and dried in a drying oven at 100 °C overnight. In the residue/analysis section, the liquid phase effluent is collected into the product barrel, and the gaseous phase can be led either to the gas analyzer (Hubei Cubic-Ruiyi Instruments CO. Ltd, Wuhan, China, Gasboard -3100 Serial Syngas Analyzer) (in the case of mixture of gases, e.g., 20% CO_2_ in N_2_) or in the flowmeter (Ritter, Bochum, Germany Gas Meter, TG1/5, 2-120 lt/h).

Benchmark tests were performed with a 100% CO_2_ stream with continuous recirculation of the CaCl_2_/NH_3_ aqueous phase, both in contactor and bubbling mode. The liquid phase was fed into the shell side, while the gas phase was fed co-currently into the lumen side. The gas flow rate was set at 0.5 L/min and liquid was recycled at a flowrate of 0.5 L/min. Table 2 summarizes the operating conditions applied for each test.

The production of CaCO_3_ particles (carbonation) was carried out by a gas-liquid reactive precipitation process. The combination of CO_2_ absorption, mixing, and chemical reaction resulted in calcium carbonate supersaturation which initiated nucleation and further crystal growth. The extent of carbonation was controlled by CaCO_3_ supersaturation. 

The precipitation reaction took place in a saturated solution of Ca^2+^ and CO_3_^2−^ ions, according to the following equation:

(1) $${\mathrm{Ca}}_{\left(\mathrm{aq}\right)}^{2+}+{\mathrm{CO}}_{3(\mathrm{aq})}^{2-}↔{\mathrm{CaCO}}_{3(s)}$$

Concerning CO_3_^2−^ ions, these were obtained once CO_2_ was dissolved in water, and the following reactions took place:

(2) $${\mathrm{CO}}_{2(g)}↔{\mathrm{CO}}_{2(l)},$$

(3) $${\mathrm{CO}}_{2(l)}+H_{2}O↔H_{2}{\mathrm{CO}}_{3},$$

(4) $$H_{2}{\mathrm{CO}}_{3}+{\mathrm{OH}}^{-}↔{\mathrm{HCO}}_{3}^{-}+H_{2}O,$$

(5) $${\mathrm{HCO}}_{3}^{-}+{\mathrm{OH}}^{-}↔{\mathrm{CO}}_{3}^{2-}+H_{2}O,$$

The overall precipitation reaction took place at room temperature in basic conditions by the addition of ammonia, according to the following equation:

(6) $${\mathrm{CaCl}}_{2}+{\mathrm{CO}}_{2}+2{\mathrm{NH}}_{4}\mathrm{OH}↔{\mathrm{CaCO}}_{3}+2{\mathrm{NH}}_{4}\mathrm{Cl}+H_{2}O,$$

Calcium carbonate consists of three distinct crystalline polymorphs with calcite (the hexagonal β-CaCO_3_) being a thermodynamically more stable form of CaCO_3_ under normal conditions [32]. Aragonite (a denser (2.83 g/cm^3^) orthorhombic λ-CaCO_3_) and vaterite (hexagonal μ-CaCO_3_) are also encountered.

In order to assess the performance of the gas-liquid contact membrane process the overall mass transfer coefficient, K_OG_, in terms of the partial pressures accounting for all mass transfer resistances (lumen, membrane, and shell side) was calculated according to the following relationships [33]:

(7) $$K_{\mathrm{OG}}=\frac{G_{\mathrm{in}}R_{g}T}{A_{m}P_{g}}\left[\left(Y_{{\mathrm{CO}}_{2}}^{\mathrm{in}}-Y_{{\mathrm{CO}}_{2}}^{\mathrm{out}}\right)-\ln\frac{Y_{{\mathrm{CO}}_{2}}^{\mathrm{out}}}{Y_{{\mathrm{CO}}_{2}}^{\mathrm{in}}}\right],$$

(8) $$Y_{{\mathrm{CO}}_{2}}=\frac{y_{{\mathrm{CO}}_{2}}}{1-y_{{\mathrm{CO}}_{2}}}\;,$$

where G_in_ is the feed mixture molar flowrate (mol/s), R_g_ is the gas constant (atm m^3^ mol^−1^ K^−1^), T is the temperature (K), A_m_ is the contact surface (m^2^) (calculated by the data from Table 1), P_g_ is the pressure (atm), and y_CO_2__ is the mole fraction of CO_2_ in order to calculate the adjusted mole fraction in the inlet ($Y_{{\mathrm{CO}}_{2}}^{\mathrm{in}}$) and the outlet ($Y_{{\mathrm{CO}}_{2}}^{\mathrm{out}}$).

### 2.3. Characterization Methods

Calcium carbonate particles were analyzed and characterized morphologically by employing a variety of analytical methods. The Particle Size Distribution (PSD) of the produced CaCO_3_ particle powders was determined by Dynamic Light Scattering (DLS, Cordouan Technologies SAS, Pessac, France). Structural parameters of the samples, such as crystallinity and average crystal size, were examined by X-Ray Diffraction (XRD D500/501, Siemens, Berlin, Germany), equipped with Cu Ka radiation source from 10–80 2θ angle with 0.04 step. The crystalline structures of carbonate samples were observed via Thermo-Gravimetric Analysis (TGA Pyris-6, Perkin Elmer, Waltham, MA, USA) and heated under 20 % O_2_ in N_2_ with a temperature increase rate of 15 °C/min from 50 °C to 600 °C, 3 °C/min from 600 °C to 800 °C, and held for 10 min at 800 °C. Morphological characterization was obtained using Scanning Electron Microscopy (SEM)/EDS (JSM-6300 JEOL Ltd., Tokyo, Japan), operating at an accelerating voltage of 20 kV and Transmission Electron Microscopy (TEM JEM 2010, JEOL Ltd., Tokyo, Japan). Chemical structural properties of calcium carbonates were confirmed via Fourier-Transform InfraRed (FTIR) spectroscopy Jasco (Jasco FTIR-6700, Tokyo, Japan) at 600–4000 cm^−1^ wavenumber.

## 3. Results

### CaCO_3_ Particles Characterization

**Figure 3:**
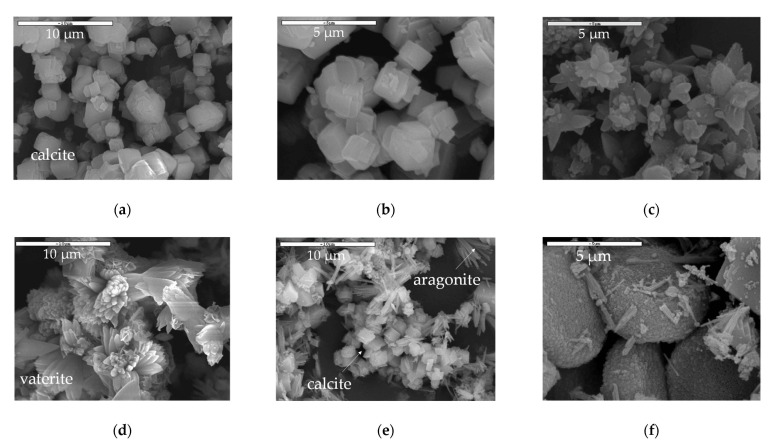
SEM analysis results of CaCO_3_ samples generated in (**a**) the contactor mode (code: 01-contactor) at lower magnification; (**b**) the contactor mode (code: 01-contactor) at higher magnification; (**c**) in the contactor mode (code: 02-contactor) at higher magnification, (**d**) the bubbling mode (code 03-bubbling) at lower magnification, (**e**) in the bubbling mode (code 04-bubbling) at lower magnification, (**f**) in the bubbling mode (code 04-bubbling) at higher magnification.

**Figure 4:**
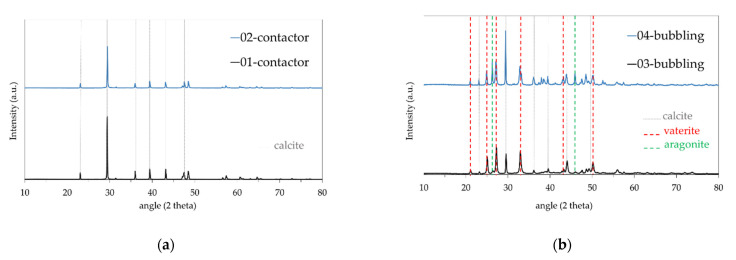
XRD analysis results for samples produced in (**a**) contactor mode; (**b**) bubbling mode.

Microstructural analysis by a combination of SEM and XRD of the contactor and bubbling samples merely revealed the calcite structure, which is the most stable CaCO_3_ crystalline phase [34] for the former (Figure 3a,b and Figure 4a) and calcite, vaterite, and aragonite structures [35] for the latter (Figure 3d–f and Figure 4b). Figure 3a,b depict the cubic calcite particles formed by the contactor mode mostly at the 1–2 μm scale, while Figure 3c,e,f show, additionally, the pine-like aragonite and flower-like vaterite morphology which form larger particles at the order of 5 μm and larger; both contactor and bubbling mode synthesized particles are consistent with the crystallite structures from the XRD analysis results depicted in Figure 4. In Figure 3e, cubic calcite particles of 1–2 μm diameter formed by the bubbling mode are also shown, while Figure 3f exhibits 5μm particle aggregates which consist of much smaller size particles. 

The calcite crystallite size, shown in Table 3, was calculated by applying the Debye-Scherrer formula on the Full Width at Half Maximum (FWHM) of the main peak at 29.42 2θ, given below:

(9) $$D_{\mathrm{hkl}}=0.89\frac{\lambda }{\beta \cos\theta }$$

The crystallite size of each sample was between 30 and 54 nm exhibiting a polycrystalline structure for all samples.

**Figure 5:**
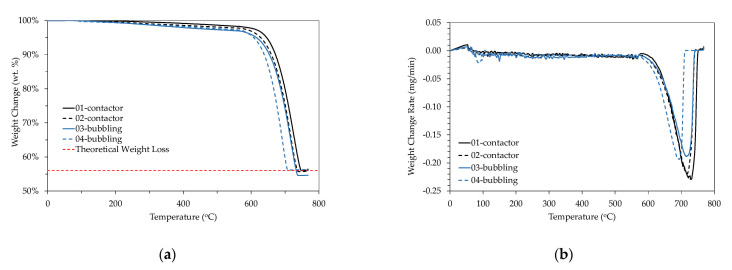
TGA analysis results (**a**) % weight change; (**b**) weight change rate.

According to the Thermo-Gravimetric Analysis (TGA) performed (Figure 5), there was a 44% weight loss for all samples in the temperature range of 680–780 °C, which is attributed to the decomposition of CaCO_3_ to CaO. All crystalline structures (calcite, aragonite, and vaterite) showed almost similar TGA patterns [36].

**Figure 6:**
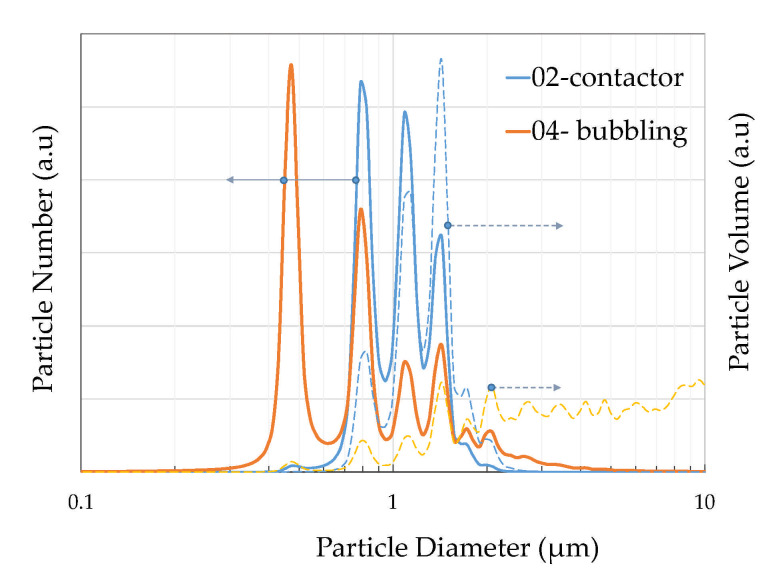
Sample PSD derived by DLS as a function of particle number (solid line right axis) and particle volume (dotted line, left axis) for the contactor and bubbling mode samples.

The DLS measurements revealed the size distribution diagrams for the two operation modes. As DLS measures particles in a suspension, the powder samples were suspended in an aqueous solution and ultrasonicated for aggregate resuspension. Figure 6 depicts the particle number distribution as a function of particle number and particle volume. Both contactor and bubbling mode particles exhibit three peaks at 786, 1037, and 1434 nm as also seen in Table 4, where the number-based diameter parameters (Dn 10%, 50%, 90%) and the volume-based diameter parameters (Dv 10%, 50%, 90%) are presented. The values were consistent with the sizes at the SEM images in Figure 3 for the calcite particles. The bubbling mode particles exhibit an additional peak at 451 nm, which can be attributed to the small calcite particles forming large aggregates in Figure 3e, which were observed mainly for the bubbling particles. The bubbling distribution also shows a shift at higher particle sizes with two additional peaks at lower particle numbers and also a gentle decrease at higher particle sizes up to 10 μm, in contrast to the contactor distribution which is eliminated roughly after 2 μm. The particle volume distribution though shows that the bubbling sample exhibits its volume capacity at much higher sizes, higher than 2 μm. This suggests that most of the sample volume consists of vaterite and aragonite particles which are characterized by higher than 2 μm sizes. It should be noted that small size particles also correspond to small volumes and vice versa, for example, 600 particles of 400 nm size may hold similar volume to one 5 μm particle, thus volume values for small particles are also respectively low. 

The sizes measured from the SEM images coincide with the size distribution diagrams of DLS in Figure 6, minimizing the agglomeration sizes observed in similar studies [16,18] due to the ultrasonicated suspension prior to measurement. EDS analysis was performed on all samples in order to detect any remnant Cl from incomplete reaction but was not measurable. The elemental analysis showed mostly Ca with a low presence of Mg (~1%).

**Figure 7:**
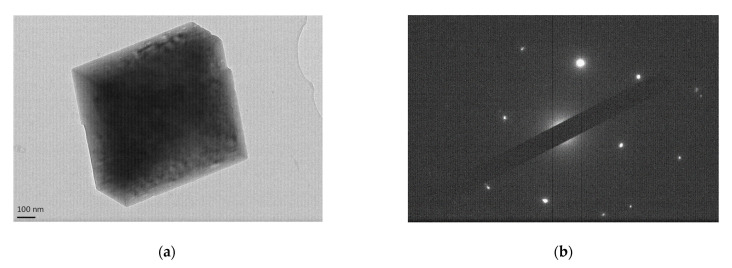
(**a**) TEM image of sample 01-contactor depicting a cubic single calcite particle and (**b**) the respective cubic diffraction pattern.

TEM images of sample 01-contactor are shown in Figure 7, showing a cubic single particle of ~750 nm of calcite and the respective diffraction pattern at Figure 7b.

**Figure 8:**
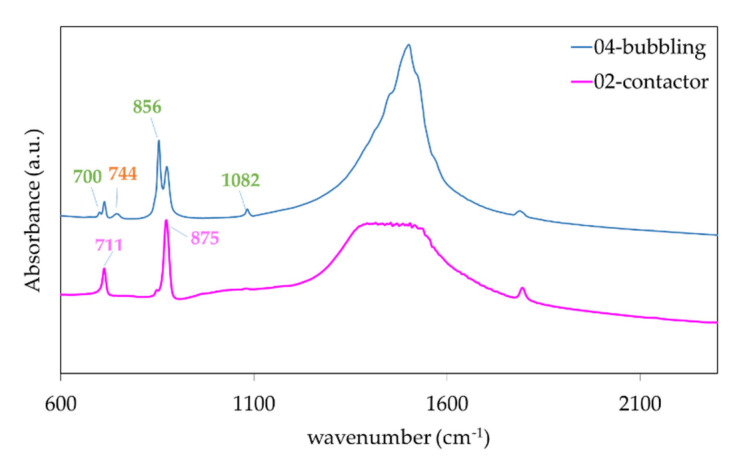
Fourier-Transform InfraRed (FTIR) spectroscopy results for samples obtained in bubbling mode (blue line) and contactor mode (pink line) exhibiting calcite, aragonite, and vaterite and only calcite peaks respectively.

Figure 8 shows the FTIR test results of three different samples. The results are indicative of pure CaCO_3_. The contactor spectrum exhibits only the characteristic calcite peaks at 711, 875, and 1435 cm^−1^, while the 04-bubbling spectrum exhibit additionally the peaks corresponding to aragonite at 700, 712, 856, 1082, 1475 and vaterite at 744, 875, 1024, 1084, 1450 cm^−1^ consistent with the crystal structures of the XRD spectrum.

Simultaneous CO_2_ capture and utilization were assessed, according to the general reaction scheme, that is, Equation (6), when the reactive solvent (aqueous solution of 885 mM CaCl_2_, 19.5 mM NH_4_OH) flows in the shell-side and the gas mixture containing 20% CO_2_ –80% N_2_ flows co-currently in the lumen-side. Gas flowrates were varied from 270 to 1400 cm^3^/min and the liquid flowrates from 165 to 440 cm^3^/min. Experiments were carried out at ambient conditions. Table 5 presents the conditions, the gas and liquid flowrates, the feed (lumen entrance), and retentate (lumen outlet) molar fractions of CO_2_ as experimentally measured, and the values for K_OG_ as calculated by Equation (7).

It is evident that the CO_2_ removal efficiency is favored by high liquid volumetric flowrates (see, e.g., No 2 and 3) for almost the same gas flowrate. Accordingly, the same liquid volumetric flowrate (see, e.g., No 3–5) cannot accommodate increasing CO_2_ volumetric flowrates resulting in smaller removal efficiencies. The overall mass transfer coefficients based on the partial pressures are always increasing when increasing either the liquid (for the same gas) volumetric flowrate (see, e.g., No 2–3), or the gas (for the same liquid) volumetric flowrate (see, e.g., No 3–5). These trends and values are in accordance with relevant reactive cases in gas-liquid membrane contact processes (see e.g., [37,38]) and indicate that both gas and liquid phase mass transfer resistances contribute to the overall mass transfer resistance.

**Figure 9:**
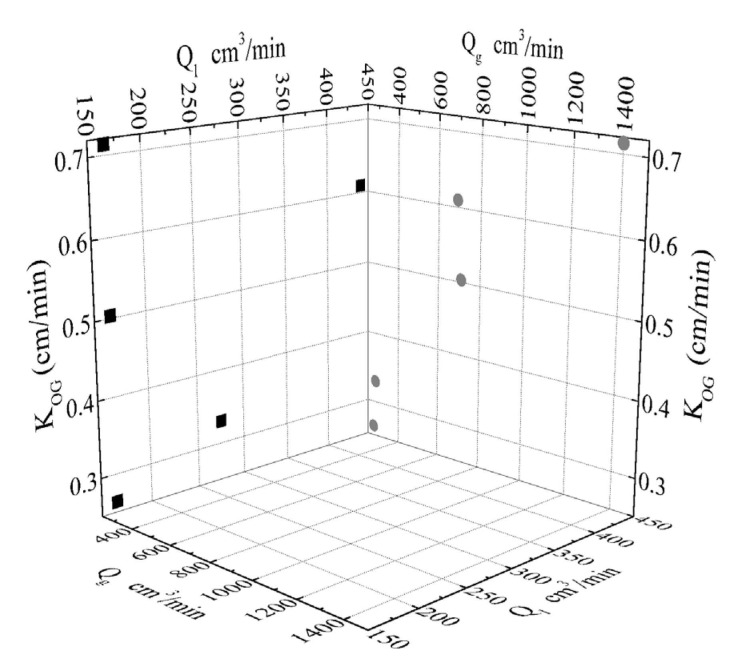
Calculated overall mass transfer coefficients (K_OG_) as a projection onto the two planes of K_OG_-Q_g_ and K_OG_-Q_l_.

The 2-D plot projections of K_OG_ values onto Q_g_ and Q_l_ planes are depicted in Figure 9. Since for the same Q_g,in_ (e.g., 270 and 280 cm^3^/min) the K_OG_ values significantly vary with altered Q_l_, it can be concluded that for the volumetric flowrates chosen and the mild concentrations of reactive solvents the liquid mass transfer resistance (shell side) significantly affects the gas-liquid contact membrane process. Respectively, for the same Q_l_ (e.g., 165.18 cm^3^/min) the K_OG_ values significantly vary with altered Q_g,in_ indicating simultaneous gas mass transfer resistance (lumen side). The above analysis in terms of the overall mass transfer coefficients hints that at the operating conditions and solution concentrations considered, the reaction regime is not an instantaneous irreversible reaction scheme, but rather a fast reaction with high CO_2_ removal capacity.

## 4. Discussion

The morphological and physicochemical characterization of the CaCO_3_ samples shows that the synthesis parameters can control the structure and size of the synthesized particles via the multiple factors that take place during synthesis, mainly the instantaneous concentrations of Ca^2+^ and CO_2_ that control the carbonation reaction. This generic observation is further supported by several published data, in which the Ca^2+^ consumption rate has been recognized to affect drastically the transition of one crystal state to another and the final formulation of lattices, regardless of the specific reactor/precipitator’s principles of operation (i.e., packed bed reactor; e.g., Murnandari et al. [15], membrane contactor/precipitator; e.g., Hosseini et al. [39], Jia et al. [40], etc.). In the current study, variations in the initial main reactant concentration, Ca^2+^, were not sought by using a broad range of the initial concentration in the solution but by employing the two operating possibilities offered by the hollow fiber porous membrane concept, that is, contactor and bubbling mode, in which “hot spots” of different degree of Ca^2+^ local excess appear.

At the contactor mode, the nominal gas-liquid interface offers a constant liquid concentration profile for Ca^2+^ reaction with CO_2_ from the gas phase, at the membrane pore openings. Therefore, nucleation time is considered to be uniform in each pore–which serves as a “hot spot” for reaction, nucleation, and crystal growth, and uniform cubic calcite particles are formed. The CO_2_ absorption rate in the aforementioned “hot spots” is affected by the uniformity in initial Ca^2+^ concentration according to remarks formulated by Jia et al. [28], who employed a semi-batch non-dispersive gas-liquid membrane contactor technique and observed that calcite was produced with a rhombohedral lattice at low initial Ca^2+^ concentrations, comparable to the one proposed in the current case. 

The dynamic bubbling reaction profile, though, promotes an instantaneous reaction on the liquid/gas interface which is initiated by nominal solution Ca^+^ concentration forming the primary nucleus, while further growth and crystallization are performed by Ca^2+^ excess in the vicinity of the decreasing bubble diameter forming vaterite and aragonite structures. Accordingly, in a recent study, Liendo et al. [16] report that principally CO_2_ bubbling offers poor control of crystal shape and size, as well as in particle size distribution, during the CaCO_3_ crystallization process in a bubbling reactor. A conventional alternative to bubbling reactor, packed bed reactor does not necessarily provide enhancement in the uniformity of crystal structure as Liendo et al. [16] suggest. Murnandari et al. [15] reported the precipitation of polymorphic forms of calcium carbonate, mainly calcite and vaterite, in a semi-batch-stirring reactor, exploiting similar conditions to the current study, that is, low initial Ca^2+^ concentration and CaCl_2_ as Ca^2+^ source. In addition, it was noticed that higher stirring speed promoted the distortion of calcite structure from cubic shape to rhombohedral and scalenohedral structures. To overcome poor control of precipitated calcium carbonate morphological characteristics, membrane-based bubbling reactor concept could be expanded to smaller membrane pore sizes, from approx. 50 nm, a pore size that corresponds to commercial polymeric hollow fibers employed throughout the literature (e.g., current study but also in [26,27,28,39,40]) to a few nanometers, approx. 1–3 nm, a pore size that corresponds to nanoporous ceramic single tube membranes. With bubbling CO_2_ through almost one order of magnitude lower pore mouths than the state-of-the-art, a transition to enhanced spatial bubbles’ distribution throughout the liquid volume is expected, while a largely increased number of carbonation reaction “hot spots” per liquid volume unit can offer advantageous mass transfer and more efficient results. 

In terms of the overall mass transfer, the performance of the gas-liquid contact membrane process was assessed and the calculations for the overall mass transfer coefficient, K_OG_, revealed a fast reaction with high CO_2_ removal capacity. Given the short duration of experiments, no reduction in overall mass transfer coefficients with time was observed, suggesting that no pore wetting was identified. Nevertheless, the liquid penetration in a hydrophobic membrane after prolonged operation may be possible (e.g., Mavroudi et al. [41]), resulting in a developing resistance in mass transfer, attributed to the membrane wetting and in a decreased CO_2_ absorption. Measures to reverse membrane wetting (e.g., post-treatment, drying, etc.), and additional approaches in gas-liquid membrane contactor operation, such as oscillating gas flow conditions proposed by Hosseini et al. [39], could improve mass transfer driving forces and enhance the general performance.

## 5. Conclusions

The gas-liquid membrane-based carbon capture and mineralization process leads to high CO_2_ recovery and the synthesis of CaCO_3_ particles, with controllable crystalline structure (aragonite, vaterite, and calcite) under both contactor and bubbling modes. Future work should focus on how the main membrane-based process parameters would affect the overall performance, as well as the morphological and structural characteristics of generated CaCO_3_ particles. Also, efforts to scale up the proposed process would further contribute to the establishment of CCUM for direct application in energy-intensive industries, for example, cement industries.

## Figures and Tables

**Table 1:** Membrane module characteristics [31].

3M™ Liqui-Cel™ MM-1 × 5.5 Series Membrane Module	Property/Value
Cartridge configuration	Parallel flow
Effective length	10 cm
Membrane material	Polypropylene
Membrane porosity	40%
Outer Diameter/Inner Diameter (OD/ID)	300 μm OD/220 μm ID
Number of fibers	2300
Module diameter	2.54 cm

**Table 2:** Experimental operating parameters of test runs.

Test No	Mode of Operation	pH Value (t = 0)	Membrane
01-contactor	Contactor	10.40	polymeric/PP ^a^
02-contactor	Contactor	10.40	polymeric/PP
03-bubbling	Bubbling	11.00	polymeric/PP
04-bubbling	Bubbling	10.60	polymeric/PP

^a^ PP: polypropylene.

**Table 3:** Calcite crystallite size of all samples.

Test No	Crystallite Size (nm)
01-contactor	47.78
02-contactor	54.15
03-bubbling	30.09
04-bubbling	54.15

**Table 4:** Number-based diameter parameters (Dn 10%, 50%, 90%) and volume-based diameter parameters (Dv 10%, 50%, 90%) of samples in contactor and bubbling mode.

Distribution Analysis	Contactor Mode (nm)	Bubbling Mode (nm)
Dn 10%	786.29	451.46
Dn 50%	1037.68	786.29
Dn 90%	1434.26	1647.66
Dv 10%	862.47	1307.57
Dv 50%	1307.57	3616.04
Dv 90%	1647.66	8704.8

**Table 5:** Experimental operating conditions and calculated CO_2_ removal efficiency and overall mass transfer coefficients.

No	T (°C)	P_g_ (bar)	Q_g,in_ (cm^3^/min)	Q_g,out_ (cm^3^/min)	Q_l_ (cm^3^/min)	CO_2_ Removal	y_CO_2_,in_ (%)	y_CO_2_,out_ (%)	K_OG_ (cm/min)
1	25.8	1.1	690	590	440.48	0.7186	19.08	6.28	0.610
2	25	1.1	280	220	275.30	0.8326	18.78	4.00	0.330
3	25.8	1.1	270	230	165.18	0.7558	18.80	5.39	0.264
4	25	1.1	710	610	165.18	0.6400	18.95	7.94	0.503
5	25	1.1	1400	1270	165.18	0.5018	19.32	10.61	0.713

## Data Availability

Data is contained within the article. Raw data are available on request from the corresponding authors and will be curated according the Data Management Plan applicable to RECODE project Grant Agreement No. 768583.
